# The fate of interneurons, GABA_A_ receptor sub‐types and perineuronal nets in Alzheimer's disease

**DOI:** 10.1111/bpa.13129

**Published:** 2022-11-21

**Authors:** Afia B. Ali, Anam Islam, Andrew Constanti

**Affiliations:** ^1^ UCL School of Pharmacy London UK

**Keywords:** Alzheimer's disease, GABA, interneurons, neurodegeneration, perineuronal nets, Synaptic

## Abstract

Alzheimer's disease (AD) is the most common neurological disease, which is associated with gradual memory loss and correlated with synaptic hyperactivity and abnormal oscillatory rhythmic brain activity that precedes phenotypic alterations and is partly responsible for the spread of the disease pathology. Synaptic hyperactivity is thought to be because of alteration in the homeostasis of phasic and tonic synaptic inhibition, which is orchestrated by the GABA_A_ inhibitory system, encompassing subclasses of interneurons and GABA_A_ receptors, which play a vital role in cognitive functions, including learning and memory. Furthermore, the extracellular matrix, the perineuronal nets (PNNs) which often go unnoticed in considerations of AD pathology, encapsulate the inhibitory cells and neurites in critical brain regions and have recently come under the light for their crucial role in synaptic stabilisation and excitatory‐inhibitory balance and when disrupted, serve as a potential trigger for AD‐associated synaptic imbalance. Therefore, in this review, we summarise the current understanding of the selective vulnerability of distinct interneuron subtypes, their synaptic and extrasynaptic GABA_A_R subtypes as well as the changes in PNNs in AD, detailing their contribution to the mechanisms of disease development. We aim to highlight how seemingly unique malfunction in each component of the interneuronal GABA inhibitory system can be tied together to result in critical circuit dysfunction, leading to the irreversible symptomatic damage observed in AD.

## 1 AN OVERVIEW OF ALZHEIMER'S DISEASE AND NETWORK DYSFUNCTION

Alzheimer's disease (AD) is the most common form of dementia and neurodegenerative condition in humans, with close to two‐thirds of all patients diagnosed with dementia being attributed to the onset of the disorder [1]. The cellular hallmarks of AD including, increased levels of both amyloid β (Aβ) deposition and neurofibrillary tangle formation, play an important function in activating neuroprotective mechanisms originating from both microglia and astrocytes, which after a certain point, begin to contribute towards the development of the pathogenesis associated with AD [2, 3]. A consistent phenotype of AD that spans from human studies to various rodent models of AD is the abnormal synaptic hyperexcitation preceding phenotypic alteration of the disease, which has been noted as a relevant therapeutic target [4, 5, 6]. Although this hyperactivity has been shown to originate in brain regions such as the lateral entorhinal cortex, which makes synaptic connections with the hippocampus, involved in factual memory storage and retrieval and is significantly affected in AD, it has been suggested that AD‐related cortical neurodegeneration is associated with the spread of this over‐excitation that propagates the pathology to other cortical regions [5, 7, 8]. This is consistent with various in vitro and in vivo models of AD, demonstrating that increased neuronal activity stimulates the release of the microtubule‐associated protein tau, which further enhances tau pathology [9, 10], as well as augmentation of Aβ depositions from presynaptic terminals [11]. Thus, there is increasing evidence to suggest abnormal proteins, Aβ and tau to be released/transferred within abnormal hyperactive synaptic circuits.

The dysfunctional inhibitory circuitry and its receptors has an emerging role in underlying the increased neuronal activity, which will take us a step closer to detecting the earliest stages of synaptic dysfunction in AD, permitting the development of novel early‐stage therapeutic interventions to stop disease progression.

## 2 INHIBITORY INTERNEURON SUBTYPES AND THEIR FATE IN AD

Pyramidal cells are the most abundant neurons of the neocortex, comprising ~80%–90% of the entire neuronal population. Interneurons containing the synthesising enzyme glutamic acid decarboxylase (GAD) for the neurotransmitter γ‐aminobutyric acid (GABA) constitute ~10%–20% of the neurons. The pyramidal cells are a relatively homogenous group when compared to interneurons, and, as such, have been classified based upon a wide range of criteria [12]. While each interneuron can perhaps be thought of as being unique, similarities in characteristics allow for the sorting of interneurons into groups. Interneurons may vary in their neuroanatomical, electrophysiological and molecular properties and fire at distinct times during network oscillations in a cell‐type‐specific manner. Each interneuron sub‐type innervates the somata, dendrites and axon initial segments of pyramidal cells to regulate their excitability and modulate the net neuronal output. Interneurons are also interconnected, where they innervate other interneurons, thus forming a dense and complex inhibitory network which is necessary for the generation of synchronised activity of large neuronal populations. This synchronous activity generates oscillations of varying frequency, associated with the execution of various hippocampal tasks [13, 14, 15].

Classifying interneurons enables groups of cells with similar properties to be linked to functional activity and allows for comparisons to be made from different studies; one particularly useful clarification of inhibitory cells is the expression of molecular markers that may be used to identify subgroups of cortical interneurons [12, 16]. In addition to their primary neurotransmitter, interneurons within the cortical regions can express neuropeptides such as cholecystokinin (CCK), somatostatin (SOM), vasoactive intestinal peptide (VIP) and neuropeptide Y (NPY). They may also contain the calcium‐binding proteins calbindin D28k (CBD), calretinin (CR) and parvalbumin (PV). These and additional proteins can be used to categorise interneuronal cells. Here, we will focus on the fate of inhibitory circuity and interneurons in AD, according to their neurochemistry.

### 2.1 Temporal and spatial resilience of Ca^2+^ binding PV‐ and CR‐expressing interneurons during AD pathogenesis

PV and CR expression can be found in sub‐classes of interneurons, predominantly neocortical basket and chandelier cells, whilst the hippocampus contains a larger variety of PV^+^ interneurons including axo‐axonic, bistratified and *oriens‐lacunosum moleculare* cells [17, 18]. Typically, PV‐expressing basket cells display the fastest‐spiking biophysical properties [19] and make synaptic contacts on postsynaptic cells proximally [20], where they have powerful inhibitory effects mediated via specific α1 subunit‐containing inhibitory GABA_A_ receptors [21]. Fast‐spiking PV interneuron dysfunction has been associated with a number of different neurological diseases including AD and various other forms of dementia as well as psychotic disorders including schizophrenia and depression [22]. Cells expressing this calcium‐binding protein are also reported to be either preserved, increased or decreased, depending on the cortical region reported in various models of AD from early and late stages of the disease [23, 24, 25, 26, 27], which could be associated with calcium homeostasis.

Any decrement in PV‐expressing interneurons in AD looks to be a process specific to the dorsal entorhinal cortex, which plays a vital function in the storage of episodic and long‐term memory, alongside the maintenance of crucial cognitive functions in the murine brain. The loss of PV interneurons here specifically, would cause hyperexcitability in pyramidal cells, leading to neuronal necrosis and a loss of potential summation [28].

We have shown a selective regional loss of PV‐expressing cells that correlated with early‐stage hyperactivity in a mouse AD model, specifically, there is a loss of PV cells in early AD in the lateral entorhinal cortex, but in CA1, PV cells remain unchanged until later stages of the disease [7]. Similar results have recently been reported in a study conducted by Umeda et al. [29] who reported a loss of PV expression in GABAergic interneurons and to amyloid precursor protein [17] impairment specifically in the dentate gyrus of mice provided with the E693Δ (Osaka) mutation, where the study found a significant reduction in both GAD67 and PV‐expression within the dentate gyrus as well as the entorhinal cortex (although the PV depletion at this particular area was non‐significant). Under these circumstances, the loss of function of PV cells was attributed to synaptic irregularities [30] in mouse homozygotes as early as 4 months. Studies have also shown that this phenomenon may not be specific to AD, since many similar neurological and psychiatric disorders including schizophrenia and autism have been reported to have a significant reduction in GAD65/67 expression located at the synaptic terminals of cerebellar PV^+^ neurones [31, 32, 33]. Although the pathology of each disease is distinctively unique, they do share certain characteristics, including the accumulation of detrimental proteins and the loss of neurons in particular positions of the brain, suggesting that the mechanism of neuronal cell death may be related in each condition [34]. One interesting theory which attempts to explain the loss of functionality in PV^+^ interneurons present in the CA1, EC and NC regions, is referred to as the Aβ ion channel hypothesis. This postulates that the build‐up of external Aβ senile plaques will eventually conclude with the integration of amyloid plaques into the neuronal membrane [35]. Once integrated into the cell membrane, the plaques are made permeable to cations, including Ca^2+^, which precedes oxidative stress and disturbed energy metabolism [36]. Evidence from this investigation supports certain claims made by this theory, since the largest accumulation of Aβ plaques in post‐phenotypic APP^NL‐F/NL‐F^ mice can be found at the boundary between the neocortex and the dorsal entorhinal cortex. Likewise, a study carried out by Garcia‐Marin et al. [37] found that most diminished GABA terminals were found to be adjacent to Aβ plaques, which supports the statement that Aβ plaque accumulation directly initiates cellular dysfunction in patients affected by AD. By applying this theory to our own findings, a valid argument can be made that Aβ plaques are directly responsible for initiating PV^+^ cell neurodegeneration by possibly allowing cells to become more permeable to Ca^2+^, as both the dorsal entorhinal cortex and neocortex are correlated with a loss of neuronal function whilst neuronal cell deficiency can be found in the dorsal entorhinal cortex.

The CR‐containing interneurons are a major part of the dis‐inhibitory network governing other inhibitory cells [38]. In contrast to PV‐expressing interneurons, have shown using a second‐generation knock‐in *APP* mouse model, that CR interneurons in AD are *preserved* anatomically and functionally, despite the presence of post‐phenotypic alterations, that is, the presence of neuroinflammation and pathological Aβ protein aggregation [39]. This specific resilience is not just limited to cells, in fact some regions like the presubiculum, also show unique ‘preserved’ morphology with an intact functional profile [40, 41].

In normal conditions, Ca^2+^ is able to regulate the cellular membrane properties via voltage‐gated Ca^2+^ channels and maintains homeostasis with other ions [42]. However, cellular membranes in AD can be altered by Aβ, which causes increasing Ca^2+^ influx and Ca^2+^‐mediated excitotoxicity [43]. Studies have reported that interneurons with calcium‐binding proteins such as CR might overcome the excitotoxicity induced by increasing intracellular Ca^2+^ concentration [44], whereas interneurons without calcium‐binding proteins but expressing neurotransmitters like CCK and SST are more likely to degenerate in AD [25].

### 2.2 Neuropeptide CCK‐ and SST‐expressing inhibitory circuitries are also vulnerable to degeneration in AD

Neuropeptide CCK‐ and SST‐expressing inhibitory interneurons display an adapting firing pattern [15], prefer to contact postsynaptic partners on proximal and distal dendrites and are responsible for fine‐tuning local circuitry mediated via α2/3‐subunit‐containing GABA_A_ receptors [21].

We and others have shown that the CCK and SST sub‐classes of interneurons are particularly vulnerable to neurodegeneration, as they are intrinsically hyperactive in the early stages of AD, and that this hyperexcitability leads to hypertoxicity [45], which is linked to infiltration of Aβ peptides [39, 46, 47]. However, whether the hyperexcitability of these cells results in Aβ production or whether Aβ infiltration in the cells results in the hyperexcited state is yet to be fully explored.

What makes these cells vulnerable to degeneration in AD could be linked to common cleavage mechanisms of the peptides in combination with toxic soluble Aβ infiltration that triggers the aggregation and degradation of Aβ, resulting in a high level of intracellular Aβ. For example, the SST neuropeptide is known as ‘amyloidogenic’, because similar cleavage processes for the formation of SST and Aβ formation from APP exist, and it is this, that is thought to contribute to the mechanisms by which SST cells degenerate, as the similar cleavage mechanisms could facilitate interactions between the two peptides before they are released from cells [48]. As a result, the normal function of SST and CCK cells is destroyed, with an outcome of cell death. Moreover, Aβ accumulation has been shown to preferentially target GABA‐producing interneurons [49]. With the important role of these interneurons in learning and memory, and the pre‐existing association of Aβ plaque deposits and neuronal death, a reduction in the density of CCK‐positive neurons would be anticipated in knock‐in AD model mice compared to wild‐type. The interaction between the CCK‐driven system and glutamatergic system, and its role in protecting neurons against the toxic effects of glutamate [50] suggests that loss of CCK‐positive interneurons may also be responsible, at least in part, for the progressive neurodegeneration observed in AD, by potentiating neuronal cell death by excitotoxicity.

## 3 ALTERATION IN GABA_A_ RECEPTOR EXPRESSION DURING THE PATHOGENESIS OF AD

GABA receptors are heterooligomeric chloride channels in the central nervous system gated by GABA. The channel is made up of five subunits selected from a pool of eight subunits with subclasses making it a total of 19 available subunit isoforms identified in the mammalian species namely, 6α, 3β, 3γ, δ, ε, π, θ and 3ρ [51] with a 20%–40% sequence identity among them [52, 53]. The availability of 19 isoforms and thus multiple conformations make this receptor extremely diverse with unique electrophysiological and pharmacological properties.

Three fundamental types of GABA receptors have been identified, GABA_A_, GABA_B_ and GABA_C_ receptors, although GABA_C_ receptors are now considered part of GABA_A_ receptors made up of only ρ subunits [54] and are mostly expressed in the retina [55]. While GABA_A_ and GABA_C_ channels are ligand‐gated ion channels, GABA_B_ receptors are exclusively made up of G‐protein‐coupled metabotropic receptors. The most common GABA_A_ receptor subtype is the α1–3, β1–3 and γ2 receptor, located synaptically. After binding with GABA, the chloride channel opens for milliseconds thus hyperpolarizing the cell membrane and preventing action potential transmission. This transient fast‐responding inhibition is also called phasic inhibition [56]. The subunits δ, ε and π replace the γ subunit in extrasynaptically located GABA receptors [57, 58]. Extrasynaptic receptors mediate a large proportion of the total GABA‐mediated inhibition and are distinguished from the synaptic receptors through their longer‐lasting chloride currents spread over a large area such as the neuron cell body as opposed to currents lasting milliseconds at single synapses [59]. This type of slow continuous inhibition, activated through ambient levels of GABA, is also called tonic inhibition. Tonic inhibition is constant over time and space and regulates a huge area, possibly a network of neurons rather than just a single cell, as opposed to phasic inhibition which relates to rapid synchronous opening of a relatively small number of GABA channels on the postsynaptic membrane within the synaptic cleft thus limiting the inhibition in time and space, in response to an action potential at a certain synapse [60].

GABA is a key player in AD pathogenesis, as multiple studies have reported lower GABA levels in the CSF and temporal cortex of AD patients, suggesting a core inhibitory dysfunction [61, 62]. In fact, almost all components of the GABAergic system in the AD brain have been shown to be negatively affected, such as GABA levels, expression levels of GABA receptors and the GABAergic neural system [63]. At the molecular level, alterations in the GABA receptor subunit composition and expression might be the puzzle piece between AD pathogenesis and GABAergic malfunction, connecting it all together.

### 3.1 Synaptic GABA_A_Rs change in AD

The subunit composition of GABA_A_ receptors is known to be severely altered in AD [63, 64, 65, 66]. However, subunits affected in AD and their expression profile remains a controversial topic. Studies show reduced expression of α1, α2, α4, δ and β2 mRNA in AD prefrontal cortex [67] and of α1, α5 and β3 mRNA in the hippocampus [66, 68]. Reduced expression of α1, α2, α5, β2, β3 and γ2 mRNA transcripts was observed in AD brains associated with decreased GABA currents in the temporal cortex [69]. Furthermore, hippocampal regions severely affected by plaques and tangles show diminished immunoreactivity for α1 and γ2, whereas an enhanced regulation of γ1 or γ3 is observed in the neuropil [70].

Tracking GABA_A_ subunit changes with neurofibrillary tangle (NFT) severity in the AD hippocampus revealed a substantial decrease in α1 protein levels and α1 and β3 mRNA levels, while β2 and β3 protein levels were more or less preserved in Braak stages five and six [68, 71]. Further studies have reported a decrease in protein and mRNA levels of the α5 subunit [65, 72] but no change in β1 protein levels with NFT progression [65]. This suggests that during NFT formation and progression, each subunit is impacted differently and undergoes unique consequences. Interestingly, cells immunopositive for the γ subunit were found to be immunonegative for NFT [70] which could point towards their protective role for cells or their loss precedes NFT formation. As discussed previously, a working hypothesis suggests that the loss of local inhibitory GABAergic networks causes excitotoxicity because of excessive Ca^2+^ influx leading to hyperphosphorylation of tau causing formation and aggregation into tangles [73, 74].

α1 and α5 have also been associated with sex‐linked changes, with strong anxiolytic‐like effects and loss of recognition memory observed in male APP^NL‐G‐F^ mice compared to females and wild‐type animals in response to low doses of diazepam [75]. This was attributed to the upregulation of α1 and α5 transcripts in the hippocampus of these animals [75] as diazepam's sedative action is mediated through the α1 subunit [76, 77] and the α5 subunit is known to develop tolerance to diazepam as evidenced in α5‐GABA_A_ receptor mutant mice [78]. This is supported in humans by another study reporting a prominent increase in α1 and α5 expression in the CA1 and CA3 regions of AD patients [64]. Kwakowsky et al. performed a comprehensive study to show cell‐layer specific alterations in α1‐3, β1‐2 and γ2 in the hippocampus, entorhinal cortex and superior temporal gyrus of AD brain tissue. They found upregulated α1 in the CA3 region and dentate gyrus but a decrease in CA1 and entorhinal cortex. A similar trend was also observed with β2 and γ2 subunits in these regions. α3 and β1 were found to be well preserved in all regions, while α2 was upregulated in *stratum oriens* of CA1‐3, *radiatum* of CA2‐3 and displayed reduced levels in CA1 *stratum pyramidale* in the AD cases [64]. Moreover, an increase in α1 labelling was mostly seen on interneuron processes and on mossy fibres projecting to the polymorphic layer of the DG in AD cases [79, 80]. This upregulation in the DG has been shown to prevent rats from acquiring epilepsy, suggesting this increase in AD might be a compensatory mechanism to the developed excitotoxicity [81]. Furthermore, it has been shown in a mouse model of epilepsy that the loss of GABAergic terminals underlies the abnormal expression of GABA_A_ receptor subunits on DG granule cell dendrites and postsynaptic neurons that contributes to neuronal hyperexcitability [81]. Also, β3 subunit expression was found to be decreased in *stratum oriens* of CA1, DG and the subiculum [64] while β2 subunit expression was upregulated in the CA2‐3 regions in the human AD brain tissue [64, 68, 82]. γ2 was upregulated in all hippocampal regions and the subiculum in AD [64]. In contrast, a decrease in mRNA content of α1, and γ2 and an increase in α2, β1, γ1 were recorded in AD human temporal cortex samples [63] and this reduction was more prominent in areas severely affected by plaques and tangles in the neuropil [63, 68]. Thus, these changes in the AD brain point towards not only compensatory mechanisms in the CNS, but they also indicate that molecular reorganisation of defined neuronal circuitry might be at play to restabilise the abnormal GABAergic inhibitory tone in the AD network. The physiological consequences of such alterations are still unknown; however, they could be implicated in cognitive and behavioural changes and evidence for these changes can be gathered from knockout studies in animal models. It was reported that a γ2 subunit heterozygous knockout mouse shows anxiety, often characterised by explicit memory bias for threat signs and harm avoidance behaviour, thus showing enhanced sensitivity for negative associations [83]. They also showed a depression‐like phenotype [84]. β3 is essential for administering the effects of general anaesthesia [85] and an α2β3γ2 combination of subunits mediates anxiolysis [76]. Thus, alterations in these subunits can cause defects in behavioural phenotypes, especially those related to depression, anxiety, amnesia and cognitive disorders as well as altering the response to drugs that target these phenotypes such as benzodiazepines. It is thus no wonder that up to 98% of AD patients experience behavioural and psychological symptoms including anxiety, hallucinations, agitation, sleep disturbances, aggression and delusions [86, 87]. Besides behavioural changes, functional alterations have also been reported in AD. Micro‐transplanted cell membranes of human temporal cortices of AD patients showed a reduction in GABA current amplitude, more profound in the younger cases [63]. This was consistent with younger patients suffering from major cortical atrophy hypometabolism and greater cognitive impairment in early onset AD patients [88, 89]. Additionally, faster desensitisation and lower GABA sensitivity was observed in AD cases which coincided with an increase in α2 and γ1 since they are less sensitive to GABA [90, 91] and in the β1 subunit, which is known to accelerate desensitisation of inhibitory postsynaptic currents in reticular thalamic neurons [92].

### 3.2 Extrasynaptic GABA_A_ receptor subtypes in AD

Beyond the synaptic fast‐acting receptors, a massive portion of the total GABA‐mediated inhibition is mediated via extrasynaptic receptors; in fact, they participate in more than 90% of the GABA‐mediated transmission [93]. These receptors have also been shown to dynamically alter cell inhibition by sensing the presynaptic GABA levels in the thalamus [94]. These receptors show little desensitisation at saturated levels of GABA, have acute sensitivity to GABA and low maximum open state probability, thus they may remain active relatively longer than the phasic γ receptors [59, 95]. Further, tonic inhibition is implicated in cognitive functions (Lee et al. 2016), neurogenesis and synaptic plasticity [96]. Extrasynaptic GABA_A_ receptors are an important element in the fabric of neuronal excitability and therefore, early modulation of these receptors could be considered an effective treatment for AD‐mediated hyperexcitability and excitotoxicity. Although GABA appears to be only a partial agonist of extrasynaptic receptors, (as they show low efficacy in the presence of GABA with *I*_MAX_ values threefold lower than the γ‐containing receptors), higher efficacy can be achieved with compounds such as THIP, gaboxadol, muscimol and neurosteroids [97]. The subunits δ, α6, α5, ρ, π are found exclusively extrasynaptically and affect slow continuous inhibition in the CNS [98]. Of the extrasynaptic receptor subunits, the δ subunit predominantly forms complexes with the α6 or α4 subunit, α4βδ receptors have been localised to thalamic relay neurons, dentate gyrus, striatal medium spiny neurons, neocortical pyramidal cells [98] while the α6βδ complex has been mapped to cerebellar granule cells [99]. The δ subunit was found to be expressed in rosette‐like inhibitory cells such as the olfactory bulb, periglomerular cells, granule cells of the cerebellar cortex and thalamocortical neurons [53], thus the long channel open times coupled with less desensitisation could lead to more sophisticated and efficient inhibitory networks in these cells. Mutations in the δ subunit have been associated with seizures in animal models and human patients [100]. Downregulation of the δ subunit has also been reported in the middle temporal gyrus tissue of AD patients studied in vitro, contributing to excitatory‐inhibitory imbalance and cognitive impairment [101]. Moreover, δ receptors along with other extrasynaptic receptors promote network shunting and reduce seizure susceptibility. Slow recovery of GABA currents shown by these receptors is important for preventing seizures and their downregulation in the dentate gyrus has been shown to produce seizure‐like events [102]. Thus, the silent epileptic activity or the development of seizures in AD patients could be attributed to downregulation of δ subunit‐containing GABA_A_ receptors, but further research is needed to elucidate the mechanisms involved.

As for α5, an increase in expression was reported in the CA1 region and downregulation in the superior temporal gyrus [64]. The α5 subunit plays a role in tonic inhibition in the CNS, has an active role in hippocampus‐dependent learning and memory [103, 104, 105, 106, 107, 108], generates gamma oscillations and regulates network excitability within the hippocampus [109, 110, 111]. While some studies have reported a decrease in α5 subunit mRNA and protein expression in the CA1‐2 regions [112, 113], other reports show preservation of the subunit in the AD hippocampus [64, 114], subiculum, entorhinal cortex and superior temporal gyrus [64], but a significant increase in the *stratum oriens* and *pyramidale* of the CA1. As a consequence, other subunits localised with α5 such as β2 and γ2 were also found to be preserved/upregulated in these regions [64]. Targeting these alterations might show some promising results, as mice with an α5 point mutation or knockout, show improved cognitive performance [104, 105]. Additionally, using inverse agonists to block α5‐containing GABA_A_ receptors shows improved cognition in rodents [106, 115], primates and humans [116]. Hence, evidence suggests that although compensatory in nature, upregulation might not be a positive feature in the case of the α5 subunit in AD.

Moreover, targeting GABA_A_ receptors has shown some interesting and promising results. Activating GABA_A_ receptors with a positive allosteric modulator in an AD mouse model for 8 weeks, decreased pathological features of AD, including Aβ production, and improved cognitive function [117]. Thus, GABA_A_ receptors are potential research and therapeutic targets in AD and are a key target for a variety of neuropsychiatric disorders such as epilepsy and anxiety [118] as well as a symptomatic target for neurodegenerative diseases such as AD.

## 4 PERINEURONAL NETS (PNNS) AND PV INTERNEURONS

PNNs, formed during development, are condensed chondroitin sulphate‐glycosaminoglycan (CS‐GAG)‐containing matrix structures in the brain, connected to a backbone of hyaluronan, and stabilised by link protein (HAPLN1) and tenascin‐R. PNNs form part of the extracellular matrix (ECM), important for providing anchorage for nerve cells and glial cells and for contributing to normal brain communication and physiology in the adult brain [119]. Development of PNNs is also classically associated with the closure of the so‐called ‘critical period’ of postnatal development during which neuronal circuits are highly plastic [120, 121, 122, 123, 124]. PNNs are built in an activity‐dependent manner, from components originating from neurons, and also from surrounding astrocytes and oligodendrocytes and are continually remodelled by secreted matrix metalloproteases (MMPs) [125]. The expression of PNNs appears to vary between and within different brain areas, including the cortex, hippocampus, amygdala, hypothalamus, basal ganglia, and cerebellum. In the cortex, they are preferentially found around the cell bodies and proximal dendrites of a population of fast‐spiking (non‐adapting) GABAergic inhibitory interneurons containing the calcium‐binding protein PV, that are important in excitatory/inhibitory balance and learning and memory [126]; (see also [127] for neurons in the medial septum/diagonal band complex). PV‐expressing interneurons are known to be highly vulnerable to stressors [128]. PNNs are thought to stabilise glutamatergic input to these neurons; thus, any disruption of PNN integrity would be expected to reduce PV+ cell excitability (and GABA release) and consequently increase target pyramidal cell excitability as seen in AD. In the hippocampus, PNNs also protect and control the of excitability of PV+ nerve cells, particularly in the hippocampal CA2 region [129, 130]. Interestingly, atypical PNNs in the CA2 of BTBR mice showing autistic‐like behaviour were recently shown to be associated with social memory dysfunction in this model [131]. Also, [132] recently reported that social memory deficits in a mouse model of AD (Tg2576) were associated with disrupted PNNs around PV+ cells in CA2 and these memory deficits and changes in PNN levels could be prevented by local injection of neuregulin‐1 (NRG‐1) (an important factor for PV cell maturation), suggesting that protecting PV cell integrity in this area may be important for retaining social memory in AD (see also [133], who suggested general targeting of PNNs for the treatment of impaired memory).

Being negatively charged, PNNs are also considered to form a protective polyanionic microenvironment around PV neurons, thus effectively acting as a cation buffer to facilitate their fast‐spiking properties [134]. They also protect them from detrimental oxidative stress [135]. Histologically, PNNs can be readily identified in brain sections by immunolabelling with Wisteria floribunda agglutinin (WFA) lectin, which specifically binds to chondroitin sulphate proteoglycans (CSPGs) (see [136, 137]).

### 4.1 PNNs are neuroprotective and may be disrupted in AD

PNNs are not only involved in regulating neuronal activity and plasticity, but also have a vital neuroprotective function. There is evidence that dysfunction in PNN structure contributes to neurological brain disorders such schizophrenia [138, 139, 140] bipolar disorder [141], epilepsy, [142], Parkinson's disease [143], as well as AD [136, 144, 145]; for reviews, see [146, 147]. There is therefore much interest in devising novel therapeutic approaches that target PNNs as a means of maintaining their role to protect normal brain physiology and overcome brain dysfunction [148].

From the available literature, the association between PNNs and AD appears to be complex and contradictory (see [149]). In fact, the different components of the ECM may have different roles to play in AD neuropathology [150]. Amyloid‐beta (Aβ) precursor protein [17] is involved in the formation of amyloid plaques, and tau protein is involved in the formation of neurofibrillary tangles. It has been suggested that PNNs provide some degree of neuroprotection from tau pathology [151] and moreover restrict tau protein dispersion and neuronal internalisation [152]. Since interneurons are completely surrounded by PNNs, they could also provide an effective protective physical barrier against Aβ neurotoxicity [153].

There is increasing evidence from rodent AD models and post‐mortem human brain tissue studies, that PNNs are extensively disrupted in AD [154]; however, aggrecan, one key component of the PNN matrix, is present in human dense‐core Aβ plaques (perhaps as a neuroprotective mechanism), and consequently, it has been shown in human cortical and subcortical AD brain tissue, that neurons associated with aggrecan‐based PNNs are somehow resistant to tau pathology [151]. Moreover, it is believed that microglia activated by neuroinflammation (by releasing ECM‐degrading proteases) [147] directly contribute to the loss of PNNs. Interestingly, aggrecan‐based PNNs were claimed to be unaffected in the brains of a transgenic mouse (Tg2576) model of AD [155]. Likewise, areas of the cortex that are rich in ECM chondroitin sulphate proteoglycans are also less disrupted in human AD tissue [136, 156]. Recently showed using the 5xFAD mouse model that chronic pharmacological depletion of microglia with colony‐stimulating factor 1 receptor inhibitor treatment (PLX5622) prevented the loss of PNNs in their model. This model is of particular interest, as it exhibits extensive Aβ plaque deposits and gliosis from 3 months of age, in the cortex and particularly, the hippocampal subiculum. The loss of PNNs has an important consequence in altering the firing behaviour of the PV+ interneurons in the areas affected, leading to local network hyperexcitability, increased seizure propensity and ultimately, cognitive deficits characteristic of AD [157, 158]; see also: [159, 160, 161, 162]. Whether PNNs are affected in human AD, however, is controversial, possibly because of procedural processing differences of post‐mortem tissue. [136] reported in their study, that AD patient brains showed significantly decreased PNN numbers and more dense‐core Aβ plaques in the cortex compared with non‐demented control brains. Overall, their study concluded that the deleterious effects of plaque accumulation in AD on PNN integrity are most likely mediated by neuroinflammation‐activated microglia. An alternative view was recently presented by Scarlett et al. [163], who suggested that PNNs are not lost but in fact remodelled in AD and other neurocognitive disorders.

### 4.2 PNN disruption in AD may affect PV cell electrophysiology

Some idea of how the disruption loss of PNNs could change the intrinsic excitability and firing properties of the enshrouded PV+ fast‐spiking interneurons can be gleaned from various specific studies, not immediately related to AD. Of particular relevance, a recent report by Stevens et al. [164] showed that in the mouse forebrain of Ank1^F/F^; Dlx^5/6^‐Cre mice, the loss of neuronal intracellular Ankyrin‐R (AnkR) (that is highly enriched in PV+ fast‐spiking interneurons), was associated with a reduction and disruption of PNNs together with a dramatic reduction in neuronal Kv3.1b (delayed rectifier‐type) K^+^ channels. As a consequence, the action potential properties of the PV+ neurons were affected and also a strong reduction in the amplitudes of action potentials towards the end of spike trains. Ankyrin‐R is a member of a family of scaffolding proteins that anchor specific ion channels, ion exchangers and ion transporters in the plasma membrane, and is encoded by the *ANK1* gene.

Interestingly, hypermethylation and reduced expression of cortical *ANK1* is associated with AD. Several studies in AD patients have described neuropathology‐associated DNA hypermethylation of *ANK1* [165, 166, 167, 168]; however, whether this hypermethylation for AnkR protein expression (which apparently can be observed in pre‐symptomatic subjects) has functional consequences for AD development is currently unknown. If the hypothesis is that dysregulation of *ANK1*, leading to reduced AnkR expression, PNN integrity and Kv3.1b channel density in PV+ cells, could be a critical pathomechanism in AD, then future strategies aimed at upregulating neuronal AnkR protein levels or PNNs could prove therapeutically beneficial.

A clue to how PNNs can maintain the fast‐firing characteristics of PV interneurons is provided by the study of [169] conducted in mouse cortical brain slices, whereby seizures induced by an implanted cranial tumour (glioma) were accompanied by degradation of (PNNs) surrounding peritumoral fast‐spiking interneurons (FSNs), resulting in a reduced firing rate of remaining cells. It was shown that the measured membrane capacitance of the FSNs was significantly increased, suggesting that PNNs have an additional physiological role as an electrostatic insulator, in reducing interneuronal membrane capacitance under normal conditions. This allows the cells to characteristically fire action potentials at high frequencies (100–800 Hz) to maintain local GABAergic inhibition. Local degradation of the PNNs was suggested to be because of tumour‐released proteolytic enzymes (MMPs), and showed that protection of the PNNs by inhibition of this proteolytic activity using the MMP inhibitor GM6001 (Ilomastat) restored normal excitability of the FSNs. This finding has some important potential therapeutic benefits for AD and possibly other neurodegenerative/neurodevelopmental diseases involving interneuronal dysfunction and excitatory/inhibitory imbalance.

Experimentally, controlled degradation of PNNs in the brain and confirmation of their relevance in maintaining the integrity and function of PV interneurons can also be accomplished by injecting a bacterial‐derived degradative enzyme, chondroitinase ABC (ChABC) directly into the brain; such protocols were described recently by Tewari et al. [170], using in situ brain slices prepared from the cerebral cortex of a mouse model of glioma‐associated epilepsy, allowing the biophysical properties of the FSNs to be assessed by patch‐clamp recordings (see also [171]). Interestingly, in two mouse AD models, ChABC injection was able to restore two different types of memory [172] also suggesting some possible therapeutic benefit from PNN protection.

Searching for other possible channel defects in AD, in the report by Verret et al. [158] using human amyloid precursor protein (hAPP) transgenic mice (which show key features of human AD), network hypersynchrony was present, associated with impaired PV cell function and decreased levels of the PV interneuron‐specific voltage‐gated sodium channel subunit Na_v_1.1. Restoring Na_v_1.1 expression levels in the hAPP mice recovered inhibitory synaptic activity and AD symptomology (see also [173]). By a similar token, specific Na_v_1.1 sodium channel activators have even been suggested to be of possible therapeutic potential for AD [174].

### 4.3 Degradation of PNNs, neuroinflammation and AD

In conclusion, we feel that there is now sufficient accumulated positive evidence to link the degradation of PNNs with neuroinflammation, microglial activation and consequent changes in numbers and firing properties of the enshrouded GABAergic PV+ interneurons in specific brain areas that are principally affected in AD. Changes in the function of these interneurons ultimately lead to an imbalance of inhibition onto principal neurons in target areas, thus hyperexcitability and spreading neurotoxicity. Experimentally, the up or down manipulations of PNNs and the resultant observed changes would seem to support this conclusion and moreover bring to light some exciting new possibilities for novel therapeutic interventions that are distinct from the current symptomatic strategies based on enhancing residual cholinergic function (donepezil, galantamine and rivastigmine) or blanket inhibition of glutamate receptors (memantine).

Just like GABA, the glutamatergic system, a major excitatory network is severely impaired in AD. Both GABA and glutamate neurotransmitter energetics are maintained by oxidative glucose metabolism which is reported to be reduced in mouse models and AD patients affecting cognition and even adult neurogenesis [175, 176]. Readers are referred to some excellent reviews on discussing the role of changes in disturbed glutamate metabolism and its effect on the GABAergic system [177, 178].

## 5 CONCLUSION

**FIGURE 1:**
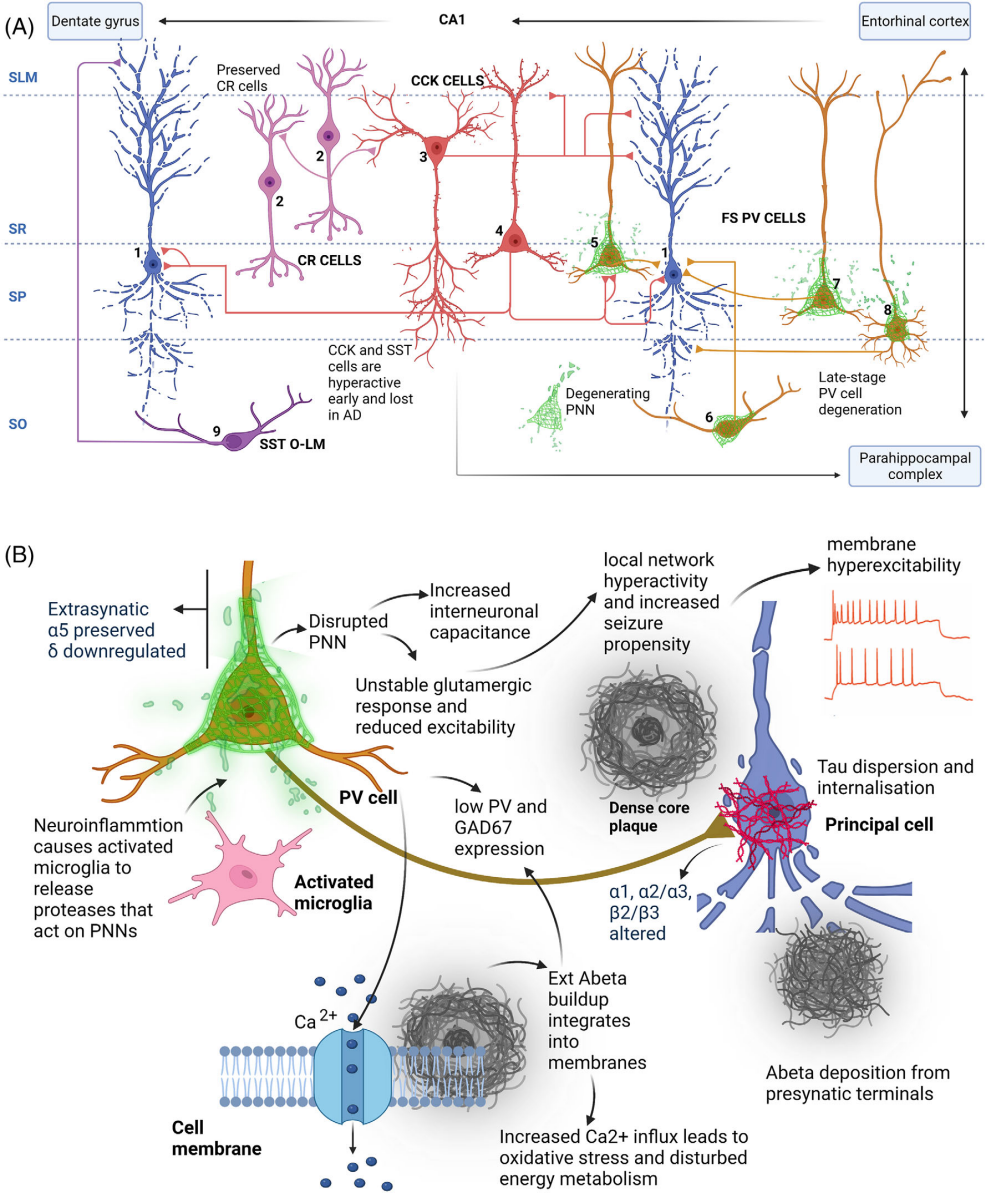
(A) Schematic overview of perisomatic and dendritic inhibitory interneurons in the hippocampal CA1 subfields that show specific regional and temporal vulnerability in AD. Examples represented are: CR interneurons (pink), CCK basket and CCK Schaffer collateral‐associated cells (red), PV basket, PV axo‐axonic, PV *oriens/lacunosum moleculare* and PV bistratified cells (yellow), and SST *oriens/lacunosum moleculare* cells (violet) with respect to CA1 subfields (dotted lines). Axonal locations are shown with respect to pyramidal cells shown in blue. CA1 layers: SLM (*stratum lacunosum moleculare*), SR (*stratum radiatum*), SP (*stratum pyramidale*) and SO (*stratum oriens*). PV cells are shown enwrapped in PNNs (green). PNNs are known to be disrupted in AD. Connections do not represent the entire inhibitory network of CA1. (B) Schematic shows suggested alterations occurring at molecular level in AD. Neuroinflammation causes active microglia (pink) to release proteases that target PNNs, destabilising PV cells. This instability manifests in reduced excitability (therefore, less GABA release), increased membrane capacitance and tau protein dispersion as well as internalisation. This leads to local network hyperexcitability, hypersynchrony, increased seizure propensity and cognitive deficits. Increased and unregulated neuronal activity is implicated in tau release causing tau pathology which reciprocally affects Aβ deposition from presynaptic terminals. Loss of PV cells as well as loss of the GABA synthetic enzyme GAD67 has been reported in AD. The external Aβ build‐up integrates into cell membranes bringing on cation permeability including Ca^**2**+^ which precedes oxidative stress and overall disturbed energy metabolism.

In summary, in this review, we describe the selective spatial and temporal vulnerability of the central inhibitory system, including GABA interneurons, their receptors and support structures during the pathogenesis of AD. Figure 1A shows a schematic of some of the major inhibitory interneurons found in the CA1 region of the hippocampus, and also throughout various cortical regions and their specific regional and temporal fate in AD. There are still missing gaps in our understanding of the status of the GABA_A_ receptors served by all of the interneurons represented during AD progression. The schematic (Figure 1B) also represents our hypothesis on the mechanisms of the Aβ/tau‐associated cascade of destruction that leads to the disruption of the PNNs and consequent PV interneuron dysfunction. The specific vulnerability of subclasses of interneurons, associated with their postsynaptic, and/or extrasynaptic GABA_A_ receptors and the PNNs have sparked recent interest, as they may be the key to targeting specific therapies to halt/prevent early and late‐stage symptoms associated with AD.

Finally, the fact that activated microglia and neuroinflammation per se may be fundamental in regulating PNN structure, we suggest that it is the early onset of neuroinflammation that needs to be primarily addressed (perhaps by selectively activating intrinsic regulatory [protective] neuronal pathways together with its causes and possible prevention/modulation in the first instance, if we are to achieve substantial changes in AD incidence and outcome in future generations of AD sufferers.

## AUTHOR CONTRIBUTIONS

Afia B. Ali designed the review and concepts and coordinated the author contributions. All authors wrote and finalised the manuscript. Anam Islam prepared the figure with advice from Afia B. Ali.

## CONFLICT OF INTEREST

The authors declare no conflict of interest.

## Data Availability

Data sharing is not applicable to this article as no new data were created or analysed in this study.
